# The Impact of Advanced Maternal Age on Pregnancy Complications and Neonatal Outcomes

**DOI:** 10.3390/jcm14155387

**Published:** 2025-07-31

**Authors:** Fikriye Karanfil Yaman, Huriye Ezveci, Sukran Dogru, Melike Sevde Harmanci, Pelin Bahçeci, Kazım Gezginç

**Affiliations:** 1 Division of Perinatology, Department of Obstetrics and Gynecology, Medical School of Meram, Necmettin Erbakan University, 42200 Konya, Turkey; huriyeezveci00@gmail.com (H.E.); melikeharmanci@gmail.com (M.S.H.); kazimgezginc@hotmail.com (K.G.); 2 Division of Perinatology, Department of Obstetrics and Gynecology, Konya City Hospital, 42020 Konya, Turkey; sukrandogru-2465@hotmail.com; 3 Department of Obstetrics and Gynecology, Medical School of Meram, Necmettin Erbakan University, 42200 Konya, Turkey; pelinbahceci30@gmail.com

**Keywords:** advanced maternal age, maternal complications, perinatal outcomes, preeclampsia, postpartum hemorrhage

## Abstract

**Objective**: This study aimed to evaluate and compare maternal and fetal outcomes between pregnancies in women aged 40 and over and those in women under 40 years of age at a tertiary care hospital. **Methods**: A retrospective cohort study was conducted at Necmettin Erbakan University Medical Faculty Hospital, analyzing data from 345 women aged 40 and over and 366 women under 40 who delivered between January 2015 and December 2024. Maternal and perinatal outcomes—including mode of delivery, gestational age, birth weight, and complications such as gestational diabetes, preeclampsia, and postpartum hemorrhage—were compared between the two groups. **Results**: Women aged 40 and over had significantly higher rates of cesarean section (73% vs. 36.1%, *p* < 0.0001), preterm delivery (27.8% vs. 18%, *p* = 0.002), and gestational diabetes (14.8% vs. 7.7%, *p* = 0.002). Additionally, these women had a higher incidence of preeclampsia (13% vs. 5.7%, *p* = 0.001) and postpartum hemorrhage (18% vs. 10.5%, *p* = 0.003). Despite these increased risks, the 5 min APGAR score was significantly higher in the ≥40 age group (median 8 vs. 7, *p* < 0.0001). The incidence of chromosomal abnormalities was significantly higher in patients≥ 40 years, with 5 cases (1.4%) reported, compared to no cases (0%) in the <40 age group (*p* = 0.025). **Conclusions**: This study shows that pregnancies in women aged 40 and above carry higher maternal and fetal risks compared to younger women. Complications such as preterm labor, cesarean delivery, gestational diabetes, and preeclampsia occur more frequently in this group. However, with careful prenatal care, positive neonatal outcomes are often achievable, highlighting the need for age-specific management and early risk detection.

## 1. Introduction

Recently, the trend toward delayed childbearing has become increasingly common, with many women choosing to have children at an older age. Although fertility generally declines with age, modern medicine has made pregnancy safer and more likely for women over 40. Advanced maternal age (AMA), typically defined as pregnancy in women 35 and older, presents unique challenges and considerations, but advanced maternal age at birth has increased today due to factors such as delayed marriage and advances in reproductive technology. Many studies on this topic classify maternal age over 35 years as advanced, while ages 40 and above are referred to as very advanced, and 45 or older as extremely advanced maternal age [1]. Additionally, women are choosing to have children later in life to focus on education and career development, which is often influenced by higher levels of education [2]. However, while birth control use is widespread, there is a significant rate of unintended pregnancies among older women due to nonuse or inconsistent use [3]. As a result of all these factors, the increasing number of pregnancies in women over 40 has highlighted the need for a more profound understanding of the medical risks and benefits associated with this demographic.

Pregnancies in women over 40 are often associated with an increased risk of chromosomal abnormalities such as Down syndrome [4], gestational diabetes [5], preeclampsia, hypertension [6], and preterm birth. As maternal age increases, the risk of congenital anomalies [7,8], low birth weight [9], and preterm birth [10] also rises.

As maternal age advances, the need for assisted reproductive techniques—particularly in vitro fertilization (IVF)—becomes more common, which in turn is associated with lower success rates and increased risk of complications [11]. Despite these challenges, many women over 40 can continue their pregnancies with the help of modern prenatal care, including improved screening techniques, genetic counseling, and better monitoring of maternal health throughout pregnancy.

This study aims to compare the maternal and fetal outcomes of pregnancies in women over the age of 40 who delivered in a tertiary care hospital with those of women under the age of 40. Although women aged 35 to 39 years may already exhibit some increased obstetric risks, we chose age 40 as the threshold for defining advanced maternal age in this study because there is a more pronounced increase in reported adverse outcomes after this point [7]. By comparing these two groups, this study aims to assess the specific effects of older maternal age on pregnancy outcomes, focusing on complications and fetal outcomes.

## 2. Materials and Methods

This retrospective study was conducted at Necmettin Erbakan University Faculty of Medicine, Obstetrics and Gynaecology, from January 2015 to December 2024, following approval from the university ethics committee (Number: 2024/4790, Application ID: 17956) and adherence to the principles outlined in the Declaration of Helsinki. In this study, we aimed to compare maternal and fetal outcomes between pregnancies in women over 40 years of age and pregnancies in women under 40 years of age. This study excluded multiple pregnancies, those with incomplete medical records, and cases with missing maternal age or other important data.

The study population was divided into two groups, as follows: the “advanced maternal age (AMA)” group, consisting of women aged 40 years and older, and the “younger maternal age” group, consisting of women aged 18 to 39 years. Initially, an equal number of participants was identified for both age groups (≥40 years and <40 years). However, after applying exclusion criteria—including multiple pregnancies, incomplete medical records, and missing data regarding maternal age or key clinical outcomes—the final sample included 345 women aged ≥40 years and 366 women aged <40 years. This minor numerical discrepancy reflects the outcome of the data cleaning process rather than any selection bias.

The primary objective was to evaluate maternal and fetal outcomes such as gestational diabetes, preeclampsia, hypertensive disorders, cesarean section rates, preterm birth, low birth weight, and fetal anomalies. Secondary outcomes included length of hospital stay, 5 min Apgar scores, and neonatal intensive care unit (NICU) admission rates.

Data on maternal age, obstetric history, comorbidities, and prenatal care were extracted from patient records. Maternal complications were defined according to standard clinical criteria, and fetal outcomes were categorized according to birth weight, gestational age at birth, and congenital anomalies identified at birth. “Normal spontaneous vaginal delivery (NSVD)” was used to refer to all vaginal births, regardless of whether labor was spontaneous or medically induced.

Statistical analysis was performed using SPSS version 25 (IBM Corp., Armonk, NY, USA). Descriptive statistics were used to summarize patient demographics, while chi-square tests and independent *t*-tests were used to compare categorical and continuous variables between the two groups. A *p*-value of less than 0.05 was considered statistically significant.

Ethical approval for the study was obtained from the hospital’s institutional review board (IRB). All information was processed following ethical guidelines for research involving human subjects.

## 3. Results

After exclusion of cases with multiple pregnancies or missing clinical data, the final study population consisted of 345 women aged ≥40 years and 366 women aged <40 years. The median age of women in group 1 was 42 years (range 40–50 years), while the median age of women in group 2 was 28 years (range 18–39 years). The median gravida (4 vs. 2, *p* < 0.0001) and parity (2 vs. 0, *p* < 0.0001) of women in group 1 were significantly higher than those in group 2. The majority of women in both groups were of Turkish ethnicity, that is, 100% in group 1 and 98.6% in group 2, with no statistically significant difference (*p* = 0.136). Mean BMI was similar between the groups, that is, 24.16 ± 5.23 kg/m^2^ in group 1 and 24.18 ± 6.49 kg/m^2^ in group 2 (*p* = 0.617). There was no statistically significant difference between the groups in terms of education level (*p* = 0.490).

The type of pregnancy determined by the use of assisted reproductive technology (ART) did not differ significantly between the two groups (5.22% in group 1 vs. 6% in group 2, *p* = 0.136). The incidence of previous cesarean delivery (CS) was similar between the two groups (*p* = 0.490); however, women in group 1 were significantly more likely to have had a previous normal spontaneous vaginal delivery (NSVD) (*p* < 0.0001). Maternal comorbidities showed several significant differences. A higher proportion of women in group 1 had a history of asthma (7.8% vs. 0.5%, *p* < 0.0001) and diabetes mellitus (4.6% vs. 1.4%, *p* = 0.006). Additionally, women in group 1 were more likely to have a history of stillbirth (5.5% vs. 1.4%, *p* = 0.004). Aspirin use was more common in women under 40 years of age (7.9% vs. 2.3%, *p* = 0.001), while thromboembolism was more common in women 40 years of age and older, although not statistically significant (3.18% vs. 1.36%, *p* = 0.147) (Table 1).

Women in group 1 had a significantly higher rate of cesarean section (73% vs. 36.1%, *p* < 0.0001) and a lower rate of normal spontaneous vaginal delivery (NSVD) (27% vs. 63.9%, *p* < 0.0001). Mean birth weight was similar between the two groups (3180 g in group 1, 3080 g in group 2, *p* = 0.648), but gestational age at delivery was significantly lower in women 40 years of age and older (37 weeks vs. 38 weeks, *p* < 0.0001). The 5 min APGAR score was significantly higher in group 1 (median 8 vs. 7, *p* < 0.0001). Fetal death was observed in six cases (1.7%) in group 1 and two cases (0.5%) in group 2; however, this difference was not statistically significant (*p* = 1.000).

The incidence of fetal anomalies did not differ significantly between the two groups (4.3% in group 1 vs. 5.5% in group 2, *p* = 0.492). The incidence of chromosomal abnormalities was significantly higher in patients aged 40 and over, with five cases (1.4%) reported, compared to none in the under-40 group (0%) (*p* = 0.025). The rate of neonatal intensive care unit (NICU) admission was higher in women aged 40 years and older (17.7% vs. 12.6%, *p* = 0.057), but this difference was not statistically significant. The gender distribution of newborns was similar between the groups (*p* = 0.400).

Adverse perinatal outcomes were more common in women aged 40 years and older. A higher proportion of women in this group developed gestational diabetes mellitus (14.8% vs. 7.7%, *p* = 0.002), preeclampsia (13% vs. 5.7%, *p* = 0.001), and preterm premature rupture of membranes (PPROM) (16.5% vs. 7.1%, *p* < 0.0001). The rate of preterm birth (<37 weeks) was significantly higher in women aged 40 years and older (27.8% vs. 18%, *p* = 0.002), and the rate of extremely preterm birth (<34 weeks) was also higher (11.3% vs. 5.73%, *p* = 0.008). Hemoglobin levels were significantly lower in group 1 during both the prepartum (12.0 g/dL vs. 12.3 g/dL, *p* < 0.0001) and postpartum periods (11.8 g/dL vs. 12.0 g/dL, *p* < 0.0001). Additionally, the rate of postpartum hemorrhage was significantly higher in group 1 (18% vs. 10.5%, *p* = 0.003). No statistically significant difference was observed in first-trimester hemoglobin levels (13 g/dL vs. 12 g/dL, *p* = 0.507). There was no statistically significant difference in postpartum transfusion rates between the two groups (7.8% vs. 10.5%, *p* = 0.194). Postpartum hospital stay longer than four days was more common among women aged 40 years and older (16.8% vs. 13.1%, *p* = 0.167), but there was no significant difference in the rate of maternal intensive care unit (ICU) admission (0.6% vs. 0%, *p* = 0.235) or postpartum hysterectomy rate (0.6% vs. 0%, *p* = 0.235) (Table 2).

## 4. Discussion

This study aimed to compare maternal and fetal outcomes in pregnancies in women aged 40 years and older with those in women under 40 years. The results of our study highlight that there are significant differences in several important maternal and perinatal factors, with older maternal age being associated with a higher risk of cesarean delivery, preterm delivery, and pregnancy-related complications (gestational diabetes, preeclampsia, postpartum hemorrhage, and PPROM), and chromosomal anomalies.

In our study, the cesarean section rate was significantly higher in group 1 (73%) compared to group 2 (36.1%) (*p* < 0.0001). In our clinic, cesarean sections were performed due to medical indications. This aligns with previous studies reporting that advanced maternal age is associated with higher cesarean rates, often due to complications like abnormal fetal presentation, prior uterine surgery, or fetal distress. In addition, physiological changes in older women, including decreased efficiency of uterine contractions, may further contribute to the increased cesarean delivery rate [12]. A study by Smithson et al. found that women of very advanced maternal age (≥45 years) had a higher cesarean section rate than the advanced maternal age group. The cesarean section rate was 69.5% in the very advanced maternal age group, compared to 39.5% in the advanced maternal age group [6]. In a systematic review by Bayrampour and Heaman, a consistent increase in cesarean birth rates was observed among older women, both nulliparous and multiparous, when compared to younger women. The risk of cesarean delivery was found to be elevated, with relative risks ranging from 1.39 to 2.76 across various studies included in the review. This relationship remains robust even after adjusting for potential confounders, suggesting an independent association between advanced maternal age and cesarean birth, although the precise mechanisms underlying this increased risk remain unclear [13].

Our findings demonstrated that women in the advanced maternal age group had a significantly higher prevalence of history of stillbirths compared to their younger counterparts. Older age is an independent risk factor for stillbirth. This is partly due to higher parity and gravidity, as well as a greater prevalence of comorbidities like hypertension and diabetes in this group. According to the *Obstetric Care Consensus No. 10* by the American College of Obstetricians and Gynecologists, advanced maternal age is among the key non-modifiable factors associated with increased stillbirth risk, alongside conditions like obesity, nulliparity, and preexisting chronic illnesses [14].

We found a higher rate of preterm premature rupture of membranes (PPROM) in women aged 40 and above, indicating increased vulnerability in this group. While this complication has been linked to various risk factors, advancing maternal age appears to play an independent role. The findings are partially supported by the study conducted by Claramonte Nieto et al., where PPROM was notably associated with women aged 45 years and older, reinforcing the idea that the likelihood of membrane rupture escalates particularly in later reproductive years [15].

Advanced maternal age is strongly linked to an increased risk of chromosomal abnormalities, especially autosomal trisomies such as trisomy 21, 18, and 13. This association is primarily attributed to meiotic errors in aging oocytes, leading to nondisjunction and resulting in chromosomal imbalance [16,17]. Additionally, some studies have reported a higher occurrence of non-chromosomal congenital anomalies with increasing maternal age. Although this association is less robust, certain structural malformations—including cardiac defects and orofacial anomalies—have been noted more frequently in older mothers [18,19,20,21]. However, these findings should be interpreted with caution, as the overall prevalence of major congenital anomalies remains low in the general population, and observed differences may reflect sample variability rather than a true causal relationship. In our study, chromosomal abnormalities were significantly more common in women aged 40 and above (1.4%, 5 cases) compared to none in younger women (0%) (*p* = 0.025). Interestingly, while chromosomal anomalies increased with age, our data did not demonstrate a significant difference between age groups in the occurrence of congenital malformations overall. This suggests that while maternal age influences chromosomal anomaly rates, its role in structural malformations may be more complex and less directly correlated.

In our study, women aged 40 years and older exhibited significantly lower hemoglobin levels in both the prepartum and postpartum periods compared to younger women. This decrease in hemoglobin may reflect age-related biological changes that begin to emerge in midlife. Age-associated increases in pro-inflammatory cytokines—such as IL-6 and TNF-α—can suppress erythropoiesis and disrupt iron metabolism via hepcidin overproduction [22,23]. In addition, clonal hematopoiesis of uncertain potential (CHIP), which begins to increase in older age, may contribute to impaired bone marrow function even in the absence of overt hematologic disease [24]. These mechanisms likely underlie the lower hemoglobin values observed in our cohort of women aged 40 and above. Furthermore, the incidence of postpartum hemorrhage (PPH) was significantly higher in this age group. Studies have shown that advanced maternal age is linked to a higher risk of postpartum hemorrhage, potentially due to age-related changes in uterine contractility and vascular health [5,25,26]. Our study found an increased incidence of postpartum hemorrhage in the older maternal age group (18% vs. 10.5%, *p* = 0.003), consistent with the literature.

The increased susceptibility to PPH among older mothers is consistent with previous research. For example, Sheen et al. reported a significantly increased rate of serious maternal complications, including PPH, in women aged 40–44 years compared to those aged 25–29 years, with an adjusted relative risk of 1.90 [27]. These results suggest that physiological changes associated with aging, such as decreased uterine contractility or vascular fragility, may compromise effective hemostasis after delivery. Similarly, Nyfløt et al. have highlighted a consistent increase in the risk of bleeding with advancing maternal age, reinforcing the importance of increased surveillance in this demographic group during the postpartum period [28].

Although our findings did not show significant differences in rates of postpartum hysterectomy or intensive care unit admission across age groups, the trend toward longer hospital stays among older women may reflect the need for extended observation and supportive care, particularly in managing anemia and maintaining hemodynamic stability. These insights highlight the need for age-adapted postpartum monitoring strategies.

In line with the literature, we also observed a significantly higher incidence of gestational diabetes mellitus (GDM) in the older age group [5,8,29]. Advanced maternal age is well known to be a risk factor for GDM due to changes in insulin sensitivity and other metabolic changes that occur with aging. As a modifiable condition, GDM represents a critical intervention point to reduce the cascade of associated complications such as hypertensive disorders and operative deliveries. While dietary modification and insulin remain the mainstays of treatment, there is growing evidence supporting the use of metformin as a safe and effective pharmacological option during pregnancy. Beyond its established role in glycemic regulation, metformin has been linked to broader metabolic and vascular benefits, and ongoing studies are investigating its use from the preconception period through postpartum, including effects on long-term maternal and child health [30]. Incorporating such targeted therapeutic strategies may significantly enhance pregnancy outcomes in AMA populations when GDM is appropriately diagnosed and managed.

Hypertension is a common pregnancy complication, with older women at higher risk. Women aged 35 and older have a significantly increased likelihood of developing chronic hypertension, with the risk rising further in those over 45 [6,31]. Additionally, the incidence of preeclampsia increases in this age group, leading to higher risks of adverse outcomes like preterm birth, small-for-gestational-age infants, and cesarean delivery [32,33]. In our study, the higher rate of preeclampsia in women over 40 years of age (13% vs. 5.7%, *p* = 0.001) supports the body of evidence linking advanced maternal age to hypertensive disorders of pregnancy [6]. This is likely due to factors such as endothelial dysfunction and an increased incidence of vascular complications in older women.

Regarding neonatal outcomes, our study showed a significantly higher incidence of preterm birth in women aged 40 years and older (27.8% vs. 18%, *p* = 0.002). This finding is consistent with previous studies showing that advanced maternal age is a risk factor for preterm birth, probably due to decreased placental function and changes in the cervix [34,35]. The study by Pinheiro et al. found that advanced maternal age (≥35 years) was associated with an increased risk of adverse obstetric and perinatal outcomes. Women aged 35–40 years and older than 40 years were more likely to have preterm birth, low birth weight, higher rates of Neonatal Intensive Care Unit (NICU) admission, and lower Apgar scores compared to women aged 20–34 years. In addition, they had higher incidences of gestational diabetes and hypertension and were more likely to have induced labor and elective cesarean delivery. The findings of this study suggest that advanced maternal age is an independent risk factor for poorer pregnancy outcomes, even in the absence of comorbidities such as gestational hypertension or diabetes [36]. Preterm birth is a significant concern as it is associated with a higher risk of neonatal morbidity and mortality. Furthermore, in our study, the rate of extremely preterm birth (<34 weeks) was significantly higher in women aged 40 years and older (11.3% vs. 5.73%, *p* = 0.008), consistent with the literature, reinforcing the need for careful monitoring of high-risk pregnancies in this age group [34].

Hochler et al. found that maternal age was strongly associated with an increased risk of adverse pregnancy outcomes. Their study showed that for each additional year of maternal age, the risk of unplanned cesarean delivery (UCD) and neonatal intensive care unit (NICU) admission was significantly increased, with a significant increase in the odds ratio (aOR) for both outcomes as maternal age increased, particularly in women aged 45–46 years. Furthermore, Hochler et al. observed that with increasing maternal age, there was an increase in adverse neonatal outcomes, including lower 5 min APGAR scores (<7), mechanical ventilation, birth asphyxia, and neonatal complications such as transient tachypnea and jaundice [37]. In our study, while higher rates of maternal and fetal complications were observed in older women, despite a slightly higher rate of NICU admission that was not statistically significant, neonatal outcomes were still relatively favorable, as indicated by significantly higher 5 min APGAR scores in women aged 40 years and older. The observation of higher 5 min APGAR scores in the advanced maternal age group, though unexpected, may reflect differences in obstetric management rather than intrinsic maternal or fetal factors. In our institution, pregnancies in older women are often carefully managed, including planned cesarean deliveries and continuous monitoring during delivery. A lower threshold for operative delivery, combined with increased intrapartum vigilance, could play a pivotal role in reducing perinatal stress and optimizing neonatal transition, as indicated by improved APGAR outcomes. Ultimately, these approaches might contribute to better neonatal adaptation at birth, as reflected by higher APGAR scores.

In the literature, women over 40 have a significantly higher risk of placenta previa, with a cohort study indicating a 10-fold increased risk compared to women aged 20–29, although the absolute risk remains low [29]. This association, however, may be partially influenced by the higher rate of previous cesarean deliveries observed in older age groups, which is a well-documented risk factor for abnormal placentation and may act as a confounding variable [38]. In our study, the incidence of placenta previa was slightly higher in women aged 40 years and over (5.5%) than in those under 40 years (2.7%), but this difference was not statistically significant (*p* = 0.062). Additionally, while the rate of placental abruption was similar in both age groups (1.4% vs. 1.1%), the literature suggests that advanced maternal age may increase the risk of placental abruption, but the effect is often confounded by factors such as multiparity and hypertension.

This study has several limitations that must be acknowledged. It was a retrospective, single-center study conducted at a tertiary university hospital primarily managing high-risk and referral cases. Therefore, the incidence of adverse maternal and perinatal outcomes in our cohort may be higher than that observed in the general obstetric population. Future prospective multicenter studies incorporating these variables are needed to better understand pregnancy outcomes at advanced maternal age.

## 5. Conclusions

This study demonstrates that pregnancies in women aged 40 and above involve greater maternal and fetal risks compared to younger women. Conditions such as preterm labor, cesarean delivery, gestational diabetes, preeclampsia, and postpartum bleeding are more frequent. As maternal age at childbirth continues to rise, it is essential to provide age-specific care plans, including early risk detection and personalized guidance. Further prospective research is necessary to better understand long-term outcomes and enhance care approaches for this population.

## Figures and Tables

**Table 1:** Comparison of demographic characteristics of the pregnancy group aged 40 and over and the pregnancy group aged under 40.

Parameters		Age 40 and over (345) Median (Min–Max)/(*n*%)	Age: Under 40 (366) Median (Min–Max)/(*n*%)	*p*-Value
Age		42 (40–50)	28 (18–39)	0.0001 ^α^
Gravida		4 (1–14)	2 (1–12)	0.0001 ^α^
Parity		2 (0–8)	0 (0–6)	0.0001 ^α^
Nationality	Turkish	345 (100%)	361 (98.6%)	0.136 ^α^
	Syria	0	2 (0.5%)	
	Other	0	3 (0.8%)	
BMI (kg/m^2^)		24.16 ± 5.23	24.18 ± 6.49	0.617 ^α^
Educational level	Primary or lower secondary	31 (9%)	21 (5.8%)	0.490 ^α^
	High school	120 (34.8%)	117 (32.1%)	
	Undergraduate	127 (36.8%)	207 (56.7%)	
	Postgraduate	67 (19.4%)	20 (5.5%)	
Conception type	Non ART	327 (94.78%)	344 (94%)	0.136 ^γ^
	ART	18 (5.22%)	22 (6%)	
Previous CS (number)		1 (0–4)	0 (0–5)	0.490 ^α^
Previous NSVD (number)		0 (0–7)	0 (0–3)	0.0001 ^α^
Maternal HT		13 (3.8%)	10 (2.7%)	0.439 ^γ^
Maternal DM		16 (4.6%)	5 (1.4%)	0.006 ^γ^
Asthma		27 (7.8%)	2 (0.5%)	0.0001 ^γ^
Autoimmune disease		44 (12.8%)	105 (28.7%)	0.0001 ^α^
History of stillbirth		19 (5.5%)	5 (1.4%)	0.004 ^γ^
Aspirin use		8 (2.3%)	29 (7.9%)	0.001 ^γ^
Thromboembolism		11 (3.18%)	5 (1.36%)	0.147 ^γ^

^α^: independent *t*-test, ^γ^: the Chi-Square test n(%), BMI: body mass index, ART: assisted reproductive treatment, C/S: cesarean section, NSVD: normal spontaneous vaginal delivery, HT: hypertension, DM: diabetes mellitus.

**Table 2:** Comparison of perinatal outcomes between pregnant women aged 40 and over and pregnant women aged under 40.

Parameters		Age 40 and over (345) Median (Min–Max)/(*n*%)	Age: Under 40 (366) Median (Min–Max)/ (*n*%)	*p*-Value
Mode of delivery	NSVD	93 (27%)	234 (63.9%)	0.0001 ^γ^
	CS	252 (73%)	132 (36.1%)	
Fetal parameters				
Birth week		37 (21–41)	38 (27–40)	0.0001 ^α^
Birth weight		3180 (340–5320)	3080 (720–5130)	0.648 ^α^
5 min APGAR		8 (0–10)	7 (0–9)	0.0001 ^α^
Stillbirth		6 (1.7%)	2 (0.5%)	1
Fetal anomaly		15 (4.3%)	20 (5.5%)	0.492 ^γ^
Chromosomal abnormalities		5 (1.4%)	0	0.025 ^γ^
Congenital malformations		10 (2.9%)	20 (5.5%)	0.086 ^γ^
NICU admission rate		61 (17.7%)	46 (12.6%)	0.057 ^γ^
Baby gender	Female	157(45.5%)	178 (48.6%)	0.400 ^γ^
	Male	188 (54.5%)	188 (51.4%)	
Adverse perinatal outcomes				
GDM		51 (14.8%)	28(7.7%)	0.002 ^γ^
GHT		10 (2.9%)	10 (2.7%)	1^γ^
PPROM		57 (16.5%)	26 (7.1%)	0.0001 ^γ^
Preeclampsia		45 (13%)	21 (5.7%)	0.001 ^γ^
Eclampsia		1 (0.3%)	0	0.485 ^γ^
HELLP		1 (0.3%)	1 (0.3%)	1^γ^
Ablatio placenta		5 (1.4%)	4 (1.1%)	0.746 ^γ^
Placenta previa		19 (5.5%)	10 (2.7%)	0.062 ^γ^
PAS		10 (2.9%)	4 (1.1%)	0.114 ^γ^
Preterm delivery (<37 weeks)		96 (27.8%)	66 (18%)	0.002 ^γ^
Preterm delivery (<34 weeks)		39 (11.3%)	21 (5.73%)	0.008 ^γ^
Hemoglobin (first trimester) (g/dL)		13 (7–15)	12 (8–15)	0.507 ^α^
Hemoglobin (prepartum) (g/dL)		12.3 (7.5–16.8)	12 (7.2–15.1)	0.0001 ^α^
Hemoglobin (postpartum) (g/dL)		11.8 (7.6–15.8)	12 (7.2–15.1)	0.0001 ^α^
Postpartum Hemorrhage		62 (18%)	38 (10.5%)	0.003 ^γ^
Postpartum hysterectomy		2 (0.6%)	0	0.235 ^γ^
Transfusion		27 (7.8%)	39 (10.7%)	0.194 ^γ^
Maternal hospital stay longer than 4 days		58 (16.8%)	48 (13.1%)	0.167 ^γ^
Maternal intensive care admission		2 (0.6%)	0	0.235 ^γ^

^α^: independent *t*-test; ^γ^: the Chi-Square test n(%); NSVD: normal spontaneous vaginal delivery; CS: cesarean section; APGAR: activity and muscle tone pulse (heart rate), grimace response (medically known as “reflex irritability”), appearance (skin coloration), respiration; NICU: neonatal intensive care unit; GDM: gestational diabetes mellitus; GHT: gestational hypertension; PPROM: preterm premature rupture of membranes; HELLP: hemolysis, elevated liver enzymes, and low platelets; PAS: placenta accreta spectrum.

## Data Availability

All authors can provide the data from the study upon reasonable request to the editorial board of the journal.
